# The Gendered Mediation Effects of Social Support on Fertility Intentions Among Childless Adults of Reproductive Age in China: National Cross-Sectional Study

**DOI:** 10.2196/77484

**Published:** 2026-02-11

**Authors:** Xinyu Xu, Peiyu Liu, Peihua Ren, Wenyan Shi, Sha Lai

**Affiliations:** 1 Health Management and Policy Institute, School of Public Policy and Administration, Xi’an Jiaotong University, No. 74, Xianning West Road, Beilin District, Xi’an, Shaanxi, 710049, China, 8602982655104; 2 Global Health Institute, Health Science Center, Xi'an Jiaotong University, Xi'an, Shaanxi, China

**Keywords:** fertility intention, social support, gender differences, self-efficacy, conscientiousness

## Abstract

**Background:**

China is currently facing a low fertility rate, making it crucial to explore the influence of psychosocial factors on fertility intentions to address demographic structural challenges. Social support, as a potentially significant influencing factor, is not yet fully understood in terms of its specific pathways and gender differences.

**Objective:**

This study aimed to explore how social support impacts fertility intentions among Chinese adults aged 20‐49 years, with an emphasis on gender-specific differences and the mediating roles of self-efficacy and conscientiousness.

**Methods:**

Data were obtained from the Psychology and Behavior Investigation of Chinese Residents (PBICR). This study included 2653 childless adults of reproductive age. A decision tree model was used to identify key factors influencing fertility intentions. A mediation analysis was conducted to explore the mediating effects of self-efficacy and conscientiousness while controlling for demographic confounders.

**Results:**

Among all 2653 participants, 71.3% (1892/2653) had fertility intentions. The proportion was significantly higher in men (weighted 79%, 95% CI 76.5%-81.3%) than in women (weighted 64.5%, 95% CI 61.8-67.1; *P*<.001). Participants with fertility intentions had a higher total social support score (mean 61.25, SD 14.02 vs mean 58.23, SD 13.01; *P*=.001). For women, family support significantly influenced fertility intentions, whereas support from friends was more relevant for men. Mediation analysis revealed that for men, self-efficacy significantly mediated the relationship between social support and fertility intention, with an indirect effect of 0.06 (95% CI 0.04-0.09; *P*=.001) and a mediation proportion of 52.54%. For women, conscientiousness played a significant mediating role, with an indirect effect of 0.011 (95% CI 0.002-0.018; *P*=.001) and a mediation proportion of 10.25%.

**Conclusions:**

Enhancing targeted social support can increase fertility intentions, with implications for addressing demographic challenges. Tailored policies should prioritize providing family support and fostering conscientiousness for women, while boosting self-efficacy and friend-based social support for men.

## Introduction

China is currently facing a profound demographic transition brought about by a low birth rate. According to the latest statistical data, the total number of births in China in 2023 was 9.02 million, with the birth rate dropping to 6.39‰ [1]. This trend has made China one of the countries with the lowest fertility rates in the world, with a total fertility rate of only 1.30 in 2020, significantly lower than the level required to maintain population replacement [2]. The low birth rate directly accelerates the process of social aging: the seventh national census shows that the proportion of people aged 65 years and older has risen from 8.92% in 2010 to 13.52%, with a total of over 190 million people [3]. To address this challenge, China has gradually relaxed birth restrictions since 2015 and implemented policies for 2 and 3 children [4]. However, policy adjustments have not brought about the expected rebound in fertility, and the trend of negative population growth continues [5]. In this context, it is urgent to delve into the influencing factors of reproductive behavior in the Chinese context.

Fertility intention refers to an individual’s expectations and attitudes toward having children within a specific period [6]. It includes two aspects: rhythm intention and quantity intention. Rhythm intention involves the timing of childbirth, while quantity intention refers to the number of children expected to be born [7]. The willingness to have children is influenced by various factors, which can be divided into micro- and macrolevels, including personal characteristics, family environment, and social influence [8]. Previous studies have shown that the economic development of a society has a huge impact on fertility intentions and that sociocultural trends also influence people’s perceptions of fertility [9]. A study of 6680 students nationwide in China showed that child health services or support were significantly associated with higher fertility intentions [10]. Thus, social support, as an important influencing factor, is gradually receiving attention.

Previous studies have shown that social support is an important factor affecting fertility intentions. It comes from emotional, informational, and instrumental assistance from family, friends, and partners and plays a crucial role in individuals’ fertility decisions [11-13]. Specifically, social support can alleviate parenting-related pressures and enhance individuals’ confidence in their parenting abilities [14]. Positive family communication and good subjective well-being have also been found to help increase the fertility intention of childless women of childbearing age [15]. However, the role of social support extends beyond its potential direct effect on fertility intentions to its mediating mechanisms. For instance, it may act as a mediator in the relationship between other psychological traits, such as self-efficacy or conscientiousness, and fertility intentions. In addition, due to the different roles and responsibilities assigned to men and women by social expectations and norms, there may be significant gender differences in the impact of social support on fertility intentions [16]. Therefore, the specific psychological pathways through which social support affects fertility intentions, and how these pathways differ between men and women, still need to be clarified through empirical research.

Therefore, this national cross-sectional study is designed to specifically investigate the psychological pathways through which social support influences fertility intentions among childless adults of reproductive age in China, with a central focus on delineating gender-specific mechanisms. It aims to (1) quantify the direct associations between multidimensional social support and fertility intentions, (2) empirically test the mediating roles of key factors within these associations, and (3) explicitly compare the strength and significance of these direct and indirect pathways between man and woman respondents.

## Methods

### Study Design and Population

Our data were derived from the large-scale cross-sectional Psychology and Behavior Investigation of Chinese Residents (PBICR). The survey used a multistage sampling design to ensure the representativeness and generalizability of the collected data. A total of 23 provinces, 5 autonomous regions, and 4 directly administered municipalities (Beijing, Tianjin, Shanghai, and Chongqing) were directly incorporated into the first stage of sampling in this study. Additionally, 2 to 6 cities in each nonprovincial capital prefecture-level administrative region of each province and autonomous region were randomly selected using a random number table, amounting to a total of 120 cities. In the second stage of sampling, a quota sampling method was used for the residents in each selected community, using quotas based on gender, age, and urban-rural distribution. The gender ratio was stipulated at 1:1, and the age distribution was similar to the age distribution in the Seventh National Population Census of China (2020).

The survey data were acquired through one-on-one interviews, using an electronic questionnaire administered via a networking questionnaire tool. The eligibility criteria for the overarching PBICR survey are shown in Textbox 1. The initial survey involved 11,031 participants.

Textbox 1. **Inclusion and exclusion criteria.**

**Inclusion criteria:**

- Age 18 years and older.
- Chinese citizenship.
- Permanent residency in China (with an annual absence of ≤1 month).
- Voluntary participation with signed informed consent.
- The ability to comprehend questionnaire items and the capacity to complete the survey independently or with noninterferential assistance.

**Exclusion criteria:**

- Impaired consciousness or psychiatric abnormalities.
- Cognitive impairment.
- Concurrent participation in comparable research.
- Unwillingness to participate.

**Figure 1.**
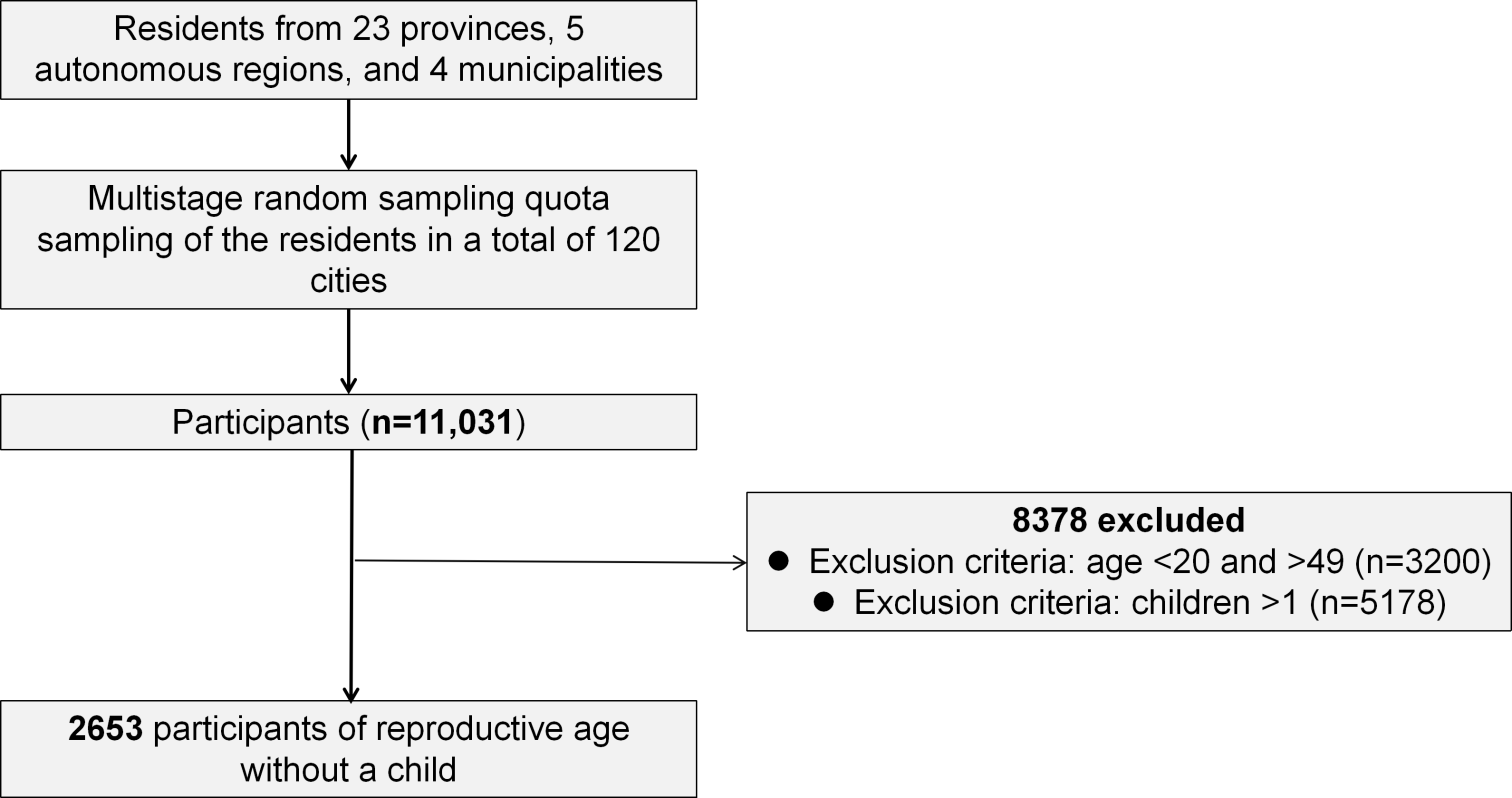
Flowchart of participant selection for the analysis of social support and fertility intentions in a national cross-sectional study.

For this analysis, we applied additional filters to this dataset to define our target subpopulation. We restricted our analysis to childless participants of childbearing age, specifically those aged 20 to 49 years. Consequently, the final analytical sample for this study comprised 2653 participants. Figure 1 shows the complete sample selection process.

**Figure 2.**
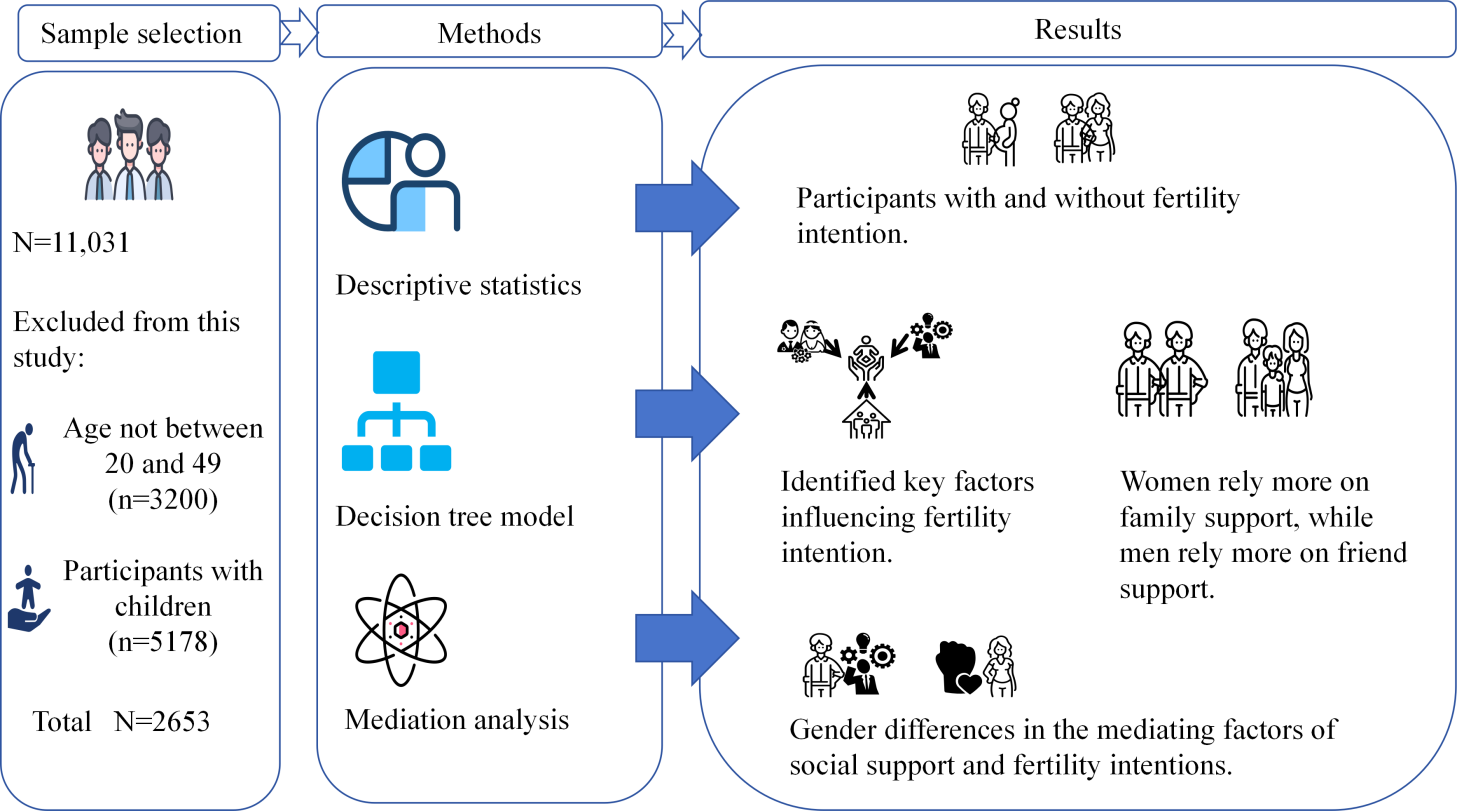
Conceptual framework of the study analyzing the gendered pathways from social support to fertility intention.

Figure 2 shows the comprehensive experimental design framework for analyzing social support and fertility intentions.

### Ethical Considerations

This study was reviewed and approved by the institutional review committee of Jinan University (approval number: JNUKY-2021-018). All procedures involving human participants were conducted in accordance with the ethical standards of this committee and with the principles of the WMA Declaration of Helsinki. Prior to participation, informed consent was obtained from all individual participants involved in the study. The consent process ensured that participants were fully informed of the study’s nature, purpose, and procedures, and they were advised that their participation was voluntary. To protect participant privacy, all collected data were anonymized at the point of entry using unique identification codes. No personally identifiable information was stored in the research databases. All electronic data were stored securely on a password-protected server with access strictly limited to authorized members of the research team.

### Outcome Variable: Fertility Intention

Fertility intention was measured using a single question, which has been widely accepted and used in demographic surveys and fertility studies [17]. The participants were asked: “How strong is your willingness to have your first child?” The response scale consisted of five options, with 1 indicating “completely unwilling,” 2 indicating “not willing,” 3 indicating “average,” 4 indicating “willing,” and 5 indicating “strongly willing.” For analysis, the fertility intention variable was used as a dichotomized variable. Participants who selected 1 or 2 were categorized as “having no intention to have children,” whereas those who selected 3, 4, or 5 were categorized as “having the intention to have children.” Unlike the descriptive analysis where the outcome was dichotomized, for the mediation analysis, fertility intention was utilized in its original 1-5 Likert scale form. It was treated as a continuous variable to facilitate linear regression modeling.

### Explanatory Variable: Social Support

The participants completed the Perceived Social Support Scale (PSSS) developed by Zimet et al [18] to assess social support. This scale comprises 12 items divided into three dimensions: (1) family support, (2) friend support, and (3) significant other support. Each item is rated on a 7-point Likert scale (1=strongly disagree to 7=strongly agree). Total scores from 12 to 36 indicate a low level of social support, scores from 37 to 60 indicate a moderate level, and scores from 61 to 84 indicate a high level of social support. We conducted separate analyses of each dimension, with each dimension consisting of 4 questions. The average score was calculated to determine the level of support for each dimension, with higher scores indicating higher levels of support. The reliability coefficient for the total social support dimension was 0.96, 0.93 for the friend support dimension, 0.90 for the significant other support dimension, and 0.90 for the family support dimension.

### Mediating Variables

The mediating variables of interest in this study were self-efficacy level, personality traits, depression symptoms, and anxiety symptoms of the respondents.

The New General Self-Efficacy Scale was used to measure the respondents’ self-efficacy level. It consists of 8 items, and each item was scored on a 5-point Likert scale (1=completely disagree, 2=disagree, 3=neutral, 4=agree, and 5=strongly agree) [19]. All items were scored positively, and the total score on the scale was calculated by summing all item scores and ranged from 8 to 40 points. The higher the score, the higher the self-efficacy level of the respondents. The Cronbach α of the scale in this study was 0.94, indicating that the scale had good reliability in this study.

The Big Five Inventory-10 (BFI-10) was used to assess the personality traits of individuals, including extraversion, agreeableness, conscientiousness, neuroticism, and openness [20,21]. This assessment uses a 5-point Likert scale, with responses ranging from 1 (completely disagree) to 5 (completely agree). A higher score in a particular personality trait indicates a stronger presence of that trait in the respondent. Numerous studies have demonstrated that the BFI-10 possesses strong reliability and validity [22,23]. In our study, the reliability of the BFI-10 was confirmed through Cronbach α analysis, yielding satisfactory results: extraversion (Cronbach α=.80), agreeableness (Cronbach α=.81), conscientiousness (Cronbach α=.86), neuroticism (Cronbach α=.79), and openness to experience (Cronbach α=.90).

The Patient Health Questionnaire-9 (PHQ-9) is a 9-item instrument used to measure the severity of depressive symptoms experienced over the past 2 weeks. Each item is rated on a scale from 0 (not at all) to 3 (nearly every day), with total scores ranging from 0 to 27 [24]. Higher scores indicate more severe depression. The PHQ-9 has been validated and shown to be reliable in various populations [25,26]. In our research, the reliability was 0.94.

The Generalized Anxiety Disorder-7 (GAD-7) tool is a 7-item scale designed to assess the severity of generalized anxiety symptoms over the previous 2 weeks. Items are scored from 0 (not at all) to 3 (nearly every day), with total scores ranging between 0 and 21. Higher scores suggest more severe anxiety [27]. The GAD-7 has demonstrated strong reliability across different groups [28,29]. In our research, the reliability was 0.96.

### Control Variables

The control variables included in this study were gender, age, BMI, marital status (married or single), rural or urban residence, number of siblings, education level (primary school or below or middle school or high school or vocational school or junior college or undergraduate or graduate or above), employment status (full-time work or part-time work or no fixed job or retirement), religious beliefs (yes or no), ethnic groups (Han or non-Han), recent medication use (none, 1, 2, 3, 4, or 5 types or more), smoking status (never or current or past), alcohol consumption (never or current or past), and annual per capita household income.

### Statistical Analysis

We constructed a decision tree model to identify the key factors influencing fertility intentions. In constructing the decision tree, the splitting criteria were based on minimizing Gini impurity, a standard measure used to quantify node homogeneity. Parameters, such as the minimum number of observations per leaf node (minsplit=20) and the maximum depth of the tree (maxdepth=5), were prespecified to prevent overfitting. These parameters were determined through cross-validation to optimize the trade-off between model complexity and predictive performance. The performance and accuracy of the decision tree model were evaluated using receiver operating characteristic (ROC) curves, which plotted the relationship between the true positive rate and the false positive rate across various classification thresholds. The area under the curve (AUC) of ROC was calculated using the *pROC* package to quantify the model’s classification performance, with values closer to 1 indicating better accuracy. We used the *rpart* package in R statistical software (version 4.4.1; R Core Team), which implements the classification and regression tree (CART) algorithm, enabling recursive partitioning of the dataset to generate interpretable decision tree models. We used mediation analysis using the *mediation* package in R to investigate the mechanisms through which independent variables influence fertility intentions through mediating variables. The parameters for the mediation model were carefully designed. Independent variables and mediators were selected based on theoretical relevance and previous empirical evidence. Continuous variables were standardized to ensure comparability across scales. The significance of the mediating effects was tested using the bootstrap method, providing robust estimates of the indirect effects. These analyses helped to elucidate the pathways through which key variables impact fertility intentions. To account for the complex sampling design and potential deviations from the population benchmarks, all analyses in this study used survey weights. The weights were constructed using a poststratification raking procedure to calibrate the sample to the national population distributions of gender, age group, and urban-rural residence among adults aged 20‐49 years, based on the 2020 Chinese National Population Census. All descriptive statistics (reported as weighted percentages or means with 95% CIs) and inferential analyses (including *χ*^2^ tests and regression models) were performed using these weights to obtain estimates that are representative of the target national subpopulation. All statistical analyses and graphing were performed using R statistical software. Numerical data were subjected to normality tests. Continuous data are presented as mean ± standard deviation, while categorical data are presented as frequency.

## Results

### Description of the Study Population

Table 1 presents the sociodemographic characteristics of the study participants based on weighted analyses to ensure national representativeness. Among the 2653 childless adults of reproductive age, an estimated 49.2% (95% CI 46.9%-51.5%) were women, and 41.2% (95% CI 38.9%-43.5%) were aged 20‐25 years. Overall, 71.3% (1892/2653) of respondents reported having fertility intentions. The fertility intention was significantly higher among man participants (weighted 79%, 95% CI 76.5%-81.3%) than among women participants (weighted 64.5%, 95% CI 61.8-67.1; *P*<.001). Marital status was also strongly associated with fertility intention (*P*<.001), with weighted 77.2% of those intending to have children being married. Other characteristics that showed significant differences between the groups with and without fertility intentions included age group (*P*=.045), monthly income (*P*=.003), number of siblings (*P*=.02), BMI (*P*<.001), smoking status (*P*=.01), and alcohol use (*P*=.04). Variables including religion, ethnicity, education, employment status, and place of residence showed no significant associations.

**Table 1.** Demographic and sociological characteristics of childless adults of reproductive age in China (N=2653).

Variable	Total (N=2653)		No fertility intention (n=761)	Having fertility intention (n=1892)	*P* value
	Unweighted n	Weighted n (95% CI)	Unweighted n (weighted %)	Unweighted n (weighted %)	
Sex					<.001
Man	1192	50.8 (48.5-53.1)	245 (33.5)	947 (49.5)	
Woman	1461	49.2 (46.9-51.5)	516 (66.5)	945 (50.5)	
Age group (years)					.045
20- 25	1301	41.2 (38.9-43.5)	388 (45.1)	913 (39.8)	
26‐30	898	35.7 (33.5-38.0)	249 (34.2)	649 (36.3)	
31‐35	286	14.1 (12.5-15.9)	63 (11.2)	223 (15.0)	
36‐40	86	5.3 (4.2-6.7)	29 (6.1)	57 (5.0)	
41‐45	53	2.5 (1.8-3.5)	23 (3.0)	30 (2.3)	
46‐49	29	1.2 (0.8-1.8)	9 (0.4)	20 (1.6)	
Marital status					<.001
Married	1930	78.5 (76.4-80.5)	420 (53.1)	1510 (77.2)	
Single	723	21.5 (19.5-23.6)	341 (46.9)	382 (22.8)	
Religion					.72
No	2585	97.5 (96.7-98.1)	744 (97.7)	1841 (97.4)	
Yes	68	2.5 (1.9-3.3)	17 (2.3)	51 (2.6)	
Ethnicity					.89
Han	2498	94.3 (93.2-95.3)	715 (94.1)	1783 (94.4)	
Other	155	5.7 (4.7-6.8)	46 (5.9)	109 (5.6)	
Education					.85
Primary school or low	35	1.5 (1.0-2.2)	13 (1.8)	22 (1.4)	
Secondary school	36	1.5 (1.1-2.1)	9 (1.3)	27 (1.6)	
High school or technical secondary	226	8.7 (7.6-10.0)	62 (8.2)	164 (8.9)	
Junior college or undergraduate	2055	77.0 (75.0-78.9)	588 (76.8)	1467 (77.1)	
Graduate or above	301	11.3 (10.0-12.7)	89 (11.9)	212 (11.0)	
Employment status					.22
Student	1441	52.1 (49.8-54.4)	439 (55.2)	1002 (51.0)	
Retired	4	0.2 (0.1-0.5)	2 (0.3)	2 (0.1)	
Employed	945	36.8 (34.7-39.0)	248 (33.1)	697 (38.1)	
Unemployed	263	10.9 (9.6-12.3)	72 (11.4)	191 (10.8)	
Monthly income, yuan					.003
≤3000	755	29.1 (27.0-31.3)	221 (30.1)	534 (28.7)	
3001‐6000	967	36.0 (33.9-38.2)	288 (37.1)	679 (35.6)	
6001‐9000	466	17.3 (15.7-19.0)	121 (15.2)	325 (18.0)	
>9000	465	17.6 (15.9-19.4)	131 (17.6)	354 (17.7)	
Residence					.22
Rural area	661	31.6 (29.4-33.9)	172 (29.8)	489 (32.2)	
Urban area	1992	68.4 (66.1-70.6)	589 (70.2)	1403 (67.8)	
Number of siblings					.02
0	1105	40.2 (38.0-42.4)	339 (42.8)	766 (39.2)	
1	1118	42.5 (40.3-44.7)	315 (41.0)	803 (43.0)	
2	284	11.3 (10.0-12.8)	75 (10.1)	209 (11.8)	
≥3	146	6.0 (5.1-7.1)	32 (6.1)	114 (6.0)	
BMI (kg/m²), mean (95% CI)	2653	21.5 (21.3-21.7)	20.9 (20.6-21.2)	21.7 (21.5-21.9)	<.001
Smoking status					.01
Never	2316	86.5 (84.8-88.0)	685 (89.1)	1631 (85.6)	
Current	242	10.1 (8.9-11.5)	48 (6.7)	194 (11.3)	
Past	95	3.4 (2.8-4.2)	28 (4.2)	67 (3.1)	
Alcohol use					.04
Never	1462	54.3 (52.0-56.6)	438 (56.8)	1024 (53.4)	
Current	773	30.5 (28.5-32.6)	193 (26.1)	580 (32.1)	
Past	418	15.2 (13.7-16.8)	130 (17.1)	288 (14.5)	

All percentages, means, and *P* values are calculated based on survey weights to ensure that the sample is consistent with the distribution of the 20‐49 age group in the 2020 Chinese National Population Census in terms of gender, age group, and urban-rural distribution. Continuous variables are represented by weighted means and their 95% CIs. The *P* value of categorical variables is based on weighted Rao Scott *χ*^2^ test, while the *P* value of continuous variables is based on weighted linear regression.

Analysis accounting for the complex survey design indicated significant associations between fertility intention and several psychosocial characteristics. Weighted scores for self-efficacy (28.85, SD 5.51 vs 27.65, SD 5.73; *P*=.001), conscientiousness (6.38, SD 1.50 vs 6.21, SD 1.53; *P*=.002), total social support (61.25, SD 14.02 vs 58.23, SD 13.01; *P*=.001), family support (4.99, SD 1.25 vs 4.85, SD 1.30; *P*=.001), significant other support (4.98, SD 1.20 vs 4.86, SD 1.27; *P*=.002), and friend support (5.09, SD 1.20 vs 5.02, SD 1.28; *P*=.049) were significantly higher in the group with fertility intention. Conversely, weighted scores for life stress (3.13, SD 1.49 vs 3.37, SD 1.46; *P*=.048) and neuroticism (5.78, SD 1.51 vs 6.12, SD 1.59; *P*=.001) were significantly lower in this group. No statistically significant differences were observed for ability to handle stress, depression, anxiety, extraversion, agreeableness, or openness. Complete data are presented in Table 2.

**Table 2.** Distribution of psychological factors, personality traits, and social support scores by fertility intention status among childless adults in China (N=2653).

Characteristic	Overall	Fertility intention		*P* value
	N=2653	Yes (n=1892)	No (n=761)	
Psychological factors				
Life stress	3.20 (1.48)	3.13 (1.49)	3.37 (1.46)	.048
Ability to handle stress	3.12 (1.65)	3.08 (1.67)	3.31 (1.61)	.08
Depression	7.43 (6.35)	7.35 (6.50)	7.72 (6.01)	.44
Anxiety	5.34 (5.26)	5.29 (5.35)	5.55 (5.04)	.60
Self-efficacy	28.48 (5.62)	28.85 (5.51)	27.65 (5.73)	.001
Personality traits				
Extraversion	6.76 (1.55)	6.71 (1.52)	6.91 (1.60)	.10
Conscientiousness	6.31 (1.51)	6.38 (1.50)	6.21 (1.53)	.002
Neuroticism	5.86 (1.54)	5.78 (1.51)	6.12 (1.59)	.001
Agreeableness	6.84 (1.52)	6.88 (1.54)	6.75 (1.48)	.06
Openness	6.19 (1.72)	6.22 (1.69)	6.16 (1.78)	.09
Social supports				
Family dimension score	4.93 (1.27)	4.99 (1.25)	4.85 (1.30)	.001
Friend dimension score	5.05 (1.23)	5.09 (1.20)	5.02 (1.28)	.049
Significant others dimension score	4.92 (1.22)	4.98 (1.20)	4.86 (1.27)	.002
Total social support score	60.38 (13.85)	61.25 (14.02)	58.23 (13.01)	.001

Data are presented as weighted mean (weighted SD). All estimates (means, SDs) and *P* values were derived from analyses that accounted for the complex, multistage sampling design by applying survey weights. The weights were constructed using poststratification raking to calibrate the sample to the national population distributions of gender, age, and urban-rural residence for adults aged 20‐49 years, based on the 2020 Chinese National Population Census. *P* values for comparisons between groups (Yes vs No fertility intention) were obtained from weighted linear regression models. The sample sizes (n) represent the unweighted number of participants in each group.

### Association Between Fertility Intentions and Social Support in the Decision Tree

**Figure 3.**
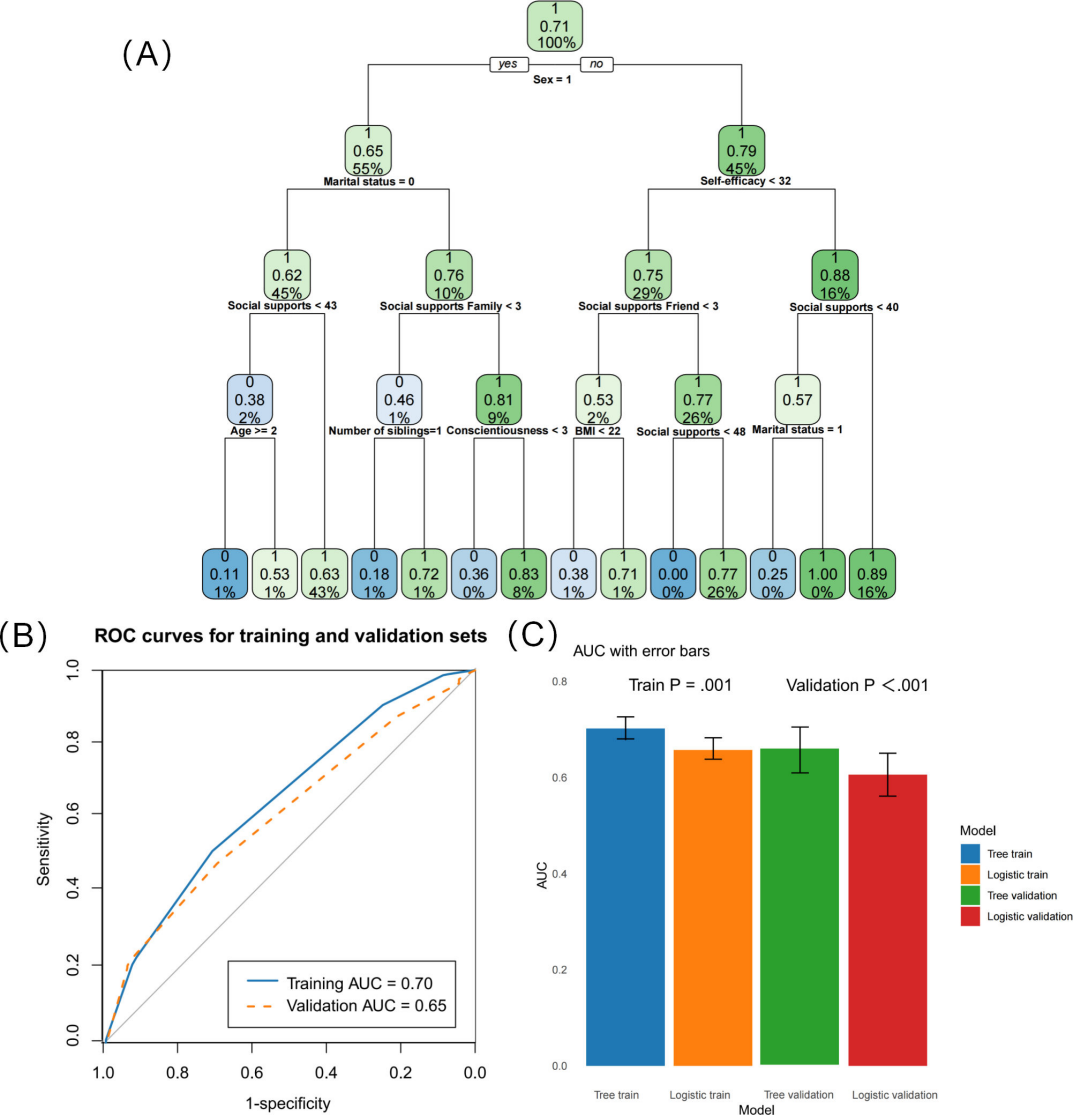
Decision tree model for predicting fertility intention and its performance evaluation among childless adults in China. (A) The DT model for predicting fertility intention. (B) ROC curves for training and validation sets. (C) AUC with error bars for training and validation sets. AUC: area under the curve; DT: decision tree; ROC: receiver operating characteristic.

The fertility intention rate of women was lower than that of men (65% vs 79%, Figure 3A). The results of our study showed that marital status was the most significant variable associated with fertility intentions among women. For women, being married and having strong family support (family support >3) was associated with a fertility intention rate of 81%. Additionally, respondents whose marital status=0 (married) and social supports of family >3 and reported Conscientiousness >3 had a higher proportion of fertility intention than married individuals with high family support but low conscientiousness (83% vs 36%). For those whose marital status was 0 but had the support of <4 family members, the proportion of fertility intention increased when the number of siblings was 1 (72% vs 18%). Our study found self-efficacy to be the strongest predictor of fertility intention among men. When self-efficacy >32 and social support >40, there was a high proportion of fertility intention (89%). Respondents with self-efficacy scores of <32, social support of friends >3, and social support >48 also presented high fertility intention rates, at 77%.

The decision tree model demonstrated strong predictive accuracy for fertility intention, with an AUC of 0.70, indicating good overall model performance. The model showed excellent diagnostic ability in both the training set (AUC=0.70) and test set (AUC=0.65), confirming its ability to generalize across different datasets (Figures 3B and 3C).

**Figure 4.**
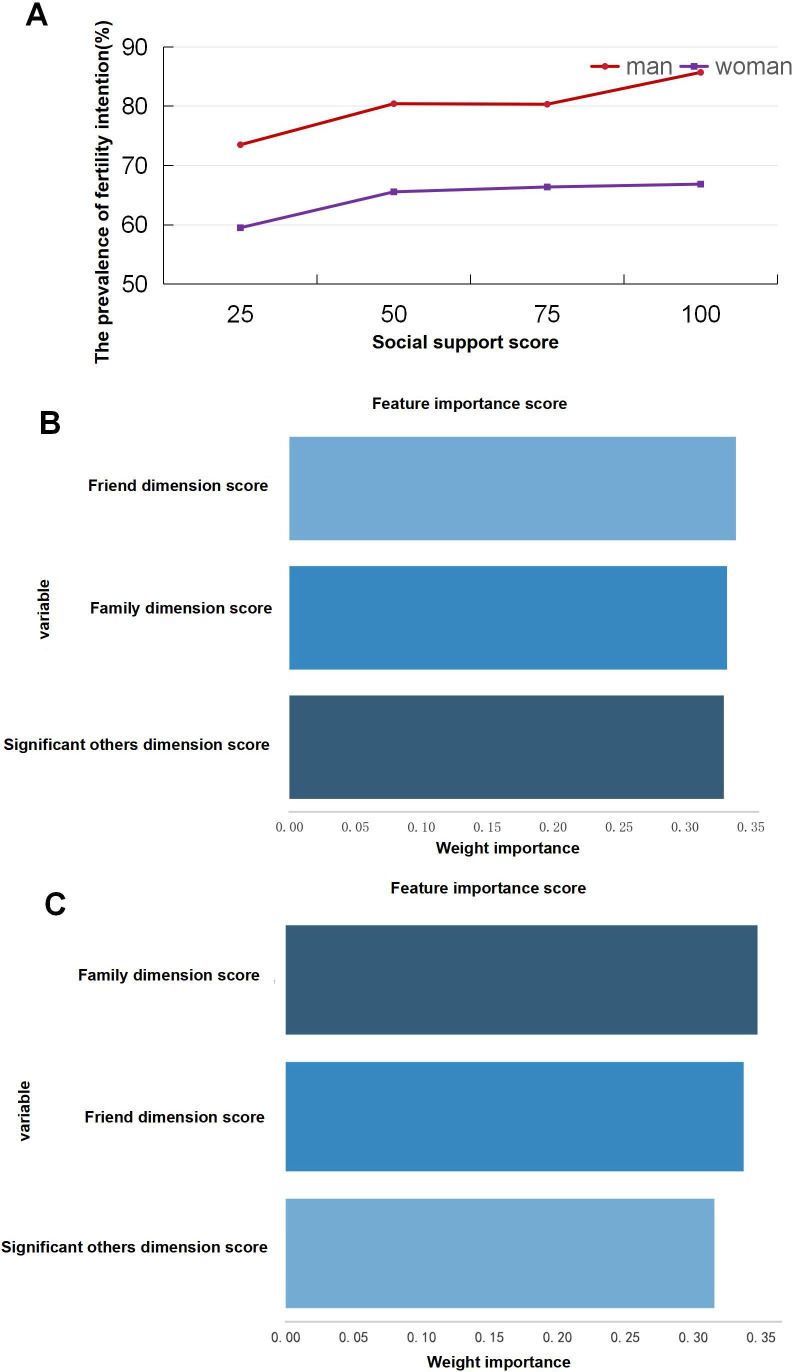
Gender differences in fertility intentions and the relative importance of different sources of social support. (A) Line chart of gender differences in fertility intentions. The horizontal axis represents the quartiles of social support, and the vertical axis represents the fertility intention percentage in this population. (B) The importance ranking of the 3 dimensions of fertility intention among men. (C) The importance ranking of the 3 dimensions of fertility intention in women.

Figure 4 indicates that increased social support correlated with higher fertility intentions for both genders, but women generally had lower intentions than men at the same support levels (Figure 4A). For women, family was the most crucial form of social support, while for men, friends held the highest importance (Figures 4B and 4C). Therefore, we explored different mediation pathways for men and women.

**Figure 5.**
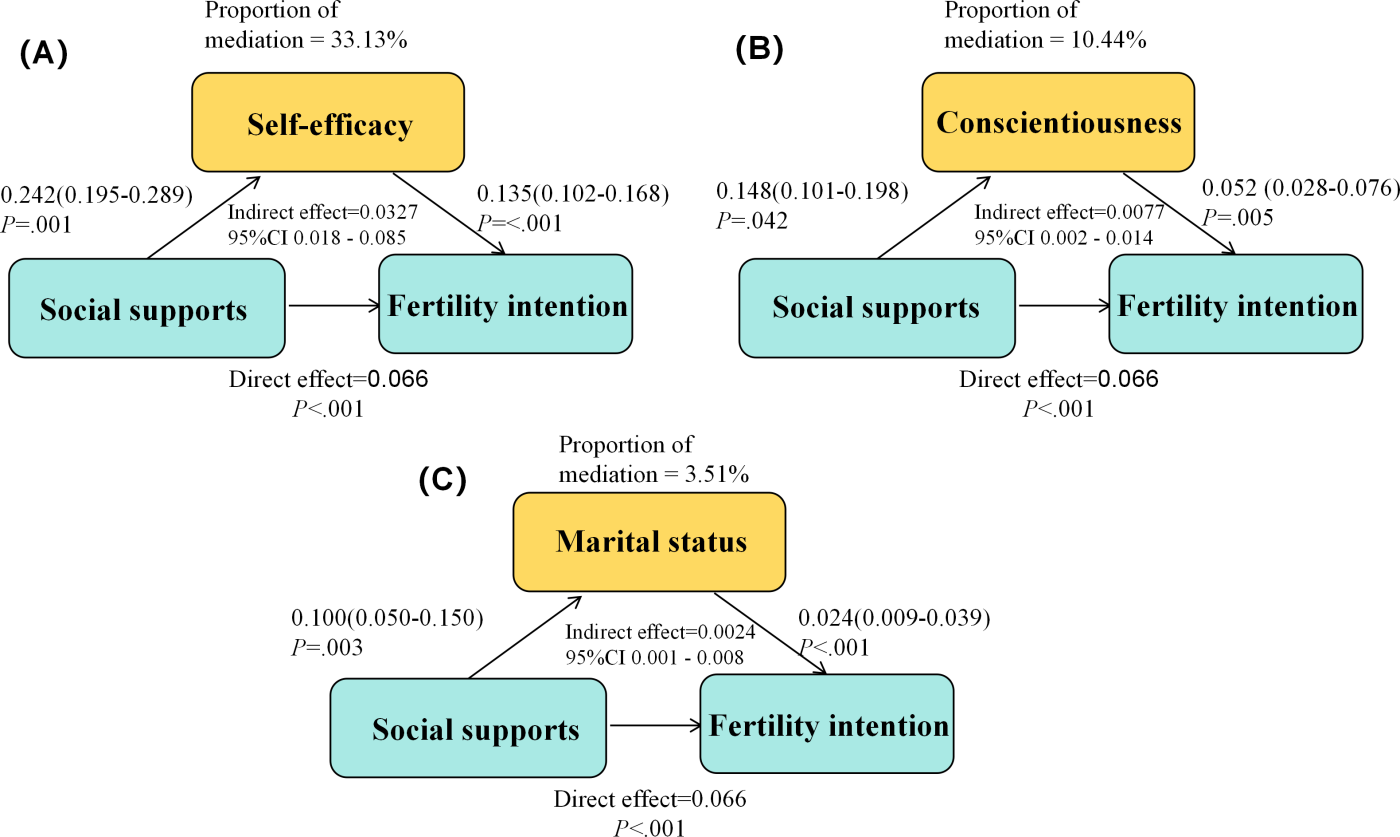
Mediation analyses examining the indirect effects of conscientiousness, self-efficacy, and marital status on the relationship between social support and fertility intention. Statistical results from mediation analyses for (A) self-efficacy (B), conscientiousness, and (C) marital status as mediators. Each panel reports the Average Causal Mediation Effect (ACME; ie, the indirect effect), the average direct effect (ADE), and the proportion of the total effect mediated, quantifying the contribution of each factor in the mediation pathway. Mediation analysis adjusted for demographic confounders

The results showed significant indirect effects of conscientiousness (mediation proportion: 10.44%), self-efficacy (mediation proportion: 33.13%), and marital status (mediation proportion: 3.51%) on the association between social support and fertility intention (Figure 5).

The mediation analysis revealed gender differences in the mediating effects of self-efficacy and conscientiousness on fertility intention (Table 3). Specifically, self-efficacy played a significant mediating role in the relationship between social support and fertility intention for men, with a mediation effect of 0.06 (95% CI 0.04 to 0.09; *P*<.001) and a mediation proportion of 52.54%. In women, self-efficacy did not demonstrate a significant mediation effect (95% CI −0.02 to 0.04; *P*=.85).

**Table 3.** Gender-stratified mediation analysis of the effect of social support on fertility intention through self-efficacy and conscientiousness among childless Chinese adults.

Exposure^a^	Mediator^b^	Outcome	Man					Woman				
			Total effect	Direct effect	Mediation effect (95% CI)	*P* value	Mediation proportion, %	Total effect	Direct effect	Mediation effect (95% CI)	*P* value	Mediation proportion, %
Social supports	Self-efficacy	Fertility intention	0.118	0.055	0.062 (0.040 to 0.087)	<.001	52.54	0.100	0.097	0.003 (−0.023 to 0.043)	.85	0
Social supports	Conscientiousness	Fertility intention	0.119	0.056	0.001 (−0.014 to 0.011)	.95	0	.100	0.089	0.011 (0.002 to 0.018)	<.001	10.25

a The Bootstrap method is used to test the mediating effect, and the 95% confidence interval (95% CI) of the mediating effect is estimated through repeated sampling (with 5000 repetitions).

b Mediation analysis adjusted for demographic confounders.

For conscientiousness, the mediation effect was significant in women, with a mediation effect of 0.011 (95% CI 0.002 to 0.018; *P*<.001) and a mediation proportion of 10.25%. In men, conscientiousness did not significantly mediate the relationship (95% CI –0.014 to 0.011, *P*=.95).

Adjustments were made for some confounding factors, including age, ethnicity, the amount of medication taken (excluding health supplements), smoking, BMI, educational level, alcohol consumption, annual income, and marital status.

## Discussion

### Principal Findings

This study examined the influence of social support on fertility intentions in Chinese adults, focusing on gender differences and the mediating effects of self-efficacy and conscientiousness, and revealed 3 key findings. First, social support was positively linked to fertility intention, suggesting that individuals with higher levels of support are more likely to express a desire to have children. Second, self-efficacy, conscientiousness, and marital status were significant mediators of the relationship between social support and fertility intention. Self-efficacy was especially relevant in men, while conscientiousness was more impactful in women. Third, a gender difference was seen in fertility intention. At the same level of social support, women had lower fertility intentions than men. However, the type of support mattered; family support was more critical for women, while men benefited significantly from friend-based support. This relationship was mediated by self-efficacy in men and conscientiousness in women. The study findings highlight important pathways for enhancing fertility intentions among men and women by enhancing social support, especially in strategy development for gender differences.

Our study revealed a positive relationship between social support and fertility intention, highlighting that individuals with stronger social networks are more likely to express a desire to have children. This finding aligns with previous research indicating that emotional and instrumental support can foster the intention to start or expand a family. Higher levels of social support, including support from family and friends, may provide the psychological and practical resources needed to manage the demands of child-rearing, thus promoting fertility intentions [30-32].

Our study identified self-efficacy, conscientiousness, and marital status as key mediators in the relationship between social support and fertility intention. Self-efficacy, which refers to one’s belief in one’s ability to achieve goals, was particularly influential for men, whereas conscientiousness, the tendency to be organized and responsible, had a stronger mediating effect in women. This supports earlier findings that personality traits and psychological factors can significantly influence fertility decisions [33]. In addition, marital status moderated this effect, with married individuals showing stronger fertility intentions than single people [30]. Together, these mediators emphasize the importance of both psychological resources and relationship status in shaping fertility intentions, providing critical pathways for interventions aimed at enhancing fertility decisions.

The study findings also highlight important gender differences in fertility intention. At equivalent levels of social support, women demonstrated lower fertility intentions than men. This phenomenon may reflect the potential influence of sociocultural and gender roles on woman fertility decisions, consistent with other studies [34-36]. Okun et al [35] suggest that Confucian culture profoundly influences China, and women take on traditional family service roles in their households. Therefore, Chinese women shoulder the dual responsibility of social work and family care and face a career fertility dilemma, an essential factor that reduces the fertility intentions of women. Furthermore, the study found that the type of social support mattered. For women, family support emerged as the most influential, while men benefited more from support from friends. These gender-specific differences in the role of support were mediated by self-efficacy in men and conscientiousness in women. These gender differences may stem from the traditional division of gender roles, where women take more responsibility and pressure in the family and, thus, are more dependent on family support when making reproductive decisions. In contrast, men are less involved in the family domain [37-39]. In many cultures, men are often expected to play the role of breadwinner and decision-maker in the family [40]. Thus, their self-efficacy is closely related to their confidence in their abilities. Social support enhances men’ sense of self-efficacy and makes them more confident in their fatherly roles and responsibilities. For women, the mediating role of responsibility between social support and fertility intentions may be related to traditional societal role expectations. Women are usually expected to take more responsibility for caring for and educating the children in the family, and therefore, their fertility intentions are closely related to their sense of responsibility [40]. Social support can help women feel more resourceful and emotionally supported, thus enhancing their sense of responsibility and making them more willing to take on the role of motherhood. Although gender roles have diversified and become more flexible with time, traditional gender role expectations still play a role.

In summary, our study findings underscore the critical role of social support in shaping fertility intentions, with self-efficacy, conscientiousness, and marital status identified as significant mediators. Importantly, gender differences were evident in how support influences reproductive decisions, with distinct pathways for men and women. This suggests that future strategies to promote fertility intentions should consider gender-specific needs and personality traits, particularly by enhancing the types of support most relevant to each gender. Policymakers and health professionals could focus on promoting family and friend support in ways that align with individual gender dynamics and personality characteristics, potentially leading to more effective fertility interventions. For example, the fertility intentions of women can be enhanced by strengthening family support and developing a sense of responsibility, whereas for men, fertility intentions can be enhanced by boosting self-efficacy and friend support.

### Limitations

However, the study has some limitations. This study has several limitations that should be considered. First, the observational cross-sectional design hinders causal inference regarding the relationships between social support, psychological mechanisms, and fertility intentions. Future longitudinal studies are needed to verify the directionality and temporal dynamics of these associations. Second, while statistically significant, some of the observed effect sizes were modest. Their practical significance should be interpreted with caution.While social support, self-efficacy, and conscientiousness are significant predictors, they account for limited variance in fertility intentions. This indicates that fertility decisions are heavily influenced by unmeasured factors beyond the psychosocial domain, such as economic constraints (e.g., housing, childcare costs) and public policies. Future research should integrate these macro-level variables to build more comprehensive models. Third, the generalizability of our findings may be limited as the study specifically focused on childless individuals of reproductive age. The applicability of the proposed model to other populations requires further examination. Finally, a key methodological limitation is the use of a single-item measure for fertility intention. Although practical in large-scale surveys, such a measure may not fully capture the complexity and multifaceted nature of reproductive planning (eg, timing and desired number of children), which could influence the depth and nuance of our conclusions.

### Conclusion

The study revealed that individuals with greater support are more likely to want children, with self-efficacy, conscientiousness, and marital status significantly influencing the link between social support and fertility intentions. Additionally, women were generally found to have lower intentions than men at the same level of social support. Family support was more crucial for women, whereas men benefited more from friend-based support. For men, this relationship was influenced by self-efficacy, whereas for women, it was affected by conscientiousness. This study enhances our understanding of gender differences in fertility intentions and offers practical guidelines for policymakers to improve these intentions by increasing social support and psychological factors.

## Abbreviations

AUC: area under the curve
BFI-10: Big Five Inventory-10
CART: classification and regression tree
GAD-7: Generalized Anxiety Disorder-7
PBICR: Psychology and Behavior Investigation of Chinese Residents
PHQ-9: Patient Health Questionnaire-9
PSSS: Perceived Social Support Scale
ROC: receiver operating characteristic
